# Stretching of memory in strategic decision making

**DOI:** 10.1186/1471-2202-13-S1-O2

**Published:** 2012-07-16

**Authors:** Alberto Bernacchia, Xiao-Jing Wang

**Affiliations:** 1 Department of Neurobiology, Yale University, USA

## 

In mixed strategy games, players make unpredictable decisions to avoid being exploited by opponents. However, human and animal subjects are often unable to make probabilistic decisions, and they cannot help but rely deterministically on past events. We studied the brain mechanisms of this fallacy in primates, and found that neural dynamics is constrained by a fundamental limitation: the integration of task events is characterized by a fixed set of timescales, and only the relative weight of different timescales can adapt to the task demands. When taking this constraint into account, the optimal strategy is to stretch memory by weighting maximally the longest timescales and counter-weighting the shorter ones. Consistently, we show that neural timescales follow the extreme-value distribution and responses display a biphasic time course. The distribution of weights predicted by the optimization process strikingly matches the experimental measurements. Our findings pose specific constraints on behavior during competitive games and highlights its underlying neural mechanisms.

